# Prevalence of Extended-Spectrum *β*-Lactamases in *E. coli* of Rats in the Region North East of Gabon

**DOI:** 10.1155/2020/5163493

**Published:** 2020-07-18

**Authors:** Richard Onanga, Pierre Philippe Mbehang Nguema, Guy Roger Ndong Atome, Arsène Mabika Mabika, Berthelemy Ngoubangoye, Wed Leslie Komba Tonda, Jean Constant Obague Mbeang, Jacques Lebibi

**Affiliations:** ^1^Laboratory of Bacteriology, International Medical Research Center of Franceville, P.O. Box 769, Franceville, Gabon; ^2^Laboratory of Research in Biochemistry, University of Science and Technology of Masuku, P.O. Box 913, Franceville, Gabon; ^3^Research Institute for Tropicale Ecology, Libreville, Gabon; ^4^Regional Graduate School of Tropical Infectious Diseases in Central Africa Franceville, P.O. Box 876, Franceville, Gabon

## Abstract

Antibiotic resistance occurs in the environment by multiplication and the spread of multidrug-resistant bacteria that would be due to an improper and incorrect use of antibiotics in human and veterinary medicine. The aim of this study was to establish the prevalence of *E.coli* producing Extended-Spectrum beta-Lactamase (ESBL) antibiotics from rats and gregarious animals in a semirural area of Gabon and to evaluate the origin of a resistance distribution in the environment from animal feces. The bacterial culture was carried out, and the identification of *E. coli* strains on a specific medium and the antibiotic susceptibility tests allowed establishing the prevalence. Characterization of resistance genes was performed by gene amplification after DNA extraction. On 161 feces collected in rats, 32 strains were isolated, and 11 strains of *E. coli* produced ESBL with a prevalence of 34.37%. Molecular tests showed that CTX-M genes 214 bp were identified in rats. The presence of CTX-M genes could have a human origin. So, the rats can carry ESBL-producing *Enterobacteriaceae* which poses a risk to human health and pets in this region of Gabon.

## 1. Introduction

The beta-lactamase family of antibiotics is widely used in the clinic. These molecules, by binding penicillin-binding protein (PBP), inhibit the synthesis of petidoglycan, an essential component the bacterial wall [1]. The first beta-lactamase plasmid (TEM-1/2, SHV-1) was initially described in the 60s in *Escherichia coli* and *Klebsiella pneumoniae* and quickly spread among other species such as *Enterobacteriaceae* [1]. But, CTX-M (Céfotaximase-Munich) diffusion mechanisms seem more complex compared to TEM (Temoneira)/SHV (sulfhydryl variable) ESBLs which is the diffusion of plasmids or other mobile genetic elements [2]. The use of extended-spectrum cephalosporin in clinical practices is at the origin of the emergence of Extended-spectrum Beta-lactamases (ESBLs) [3]. They are the consequence of theurapeutic failures [4, 5]. Antibiotic resistance has become a public health problem and has led to an increased mortality in the human population [6]. In 2018, the World Health Organization estimated that 500,000 people had been suspected of bacterial infections in 22 countries [7].

*Enterobacteriaceae* producing ESBL are a major cause of resistance to penicillin, cephalosporin, carbapenenes, and aztreonam [8]. *Enterobacteriaceae* in the gut of humans and animals acquire resistance through the selection pressure of antibiotics and their ability to exchange genetic material [9]. Thus, the spread of ESBL strains of Enterobacteria in the environment and wildlife has been observed [10–12].

Some authors around the world, and also, in Africa and many other countries around the world, have studied the spread of antibiotic-resistant bacteria in wildlife [10, 11] and urban environment [13]. Some studies consider wildlife as a potential reservoir of antibiotic resistance [14–18]. Furthermore, the prevalence of antibiotic resistance in wildlife is thought to decrease progressively with increasing distance from humans [10, 11].

Rats are known to be vectors of a variety of zoonotic pathogens responsible for significant human morbidity and mortality in cities around the world [19, 20]. Also, antibiotic-resistant *Enterobacteriaceae* have been isolated from urban rats [21–26]. Rats and other animals constitute a sentinel of choice for studies on the spread of resistance in community and wilderness [23, 27].

Gabon has a very diverse fauna, rats are well represented on the territory, and several studies on the carriage of different pathogens have already been carried out [28–30]. There are no data on the prevalence of antibiotic-resistant bacteria in rats in Gabon. The aim of this study is to evaluate the prevalence of ESBLs in Enterobacteria isolated from rat feces in the urban area of Makokou (Ogooue Ivindo province, Gabon).

## 2. Materials and Methods

### 2.1. Sampling Period and Site

The sampling was performed in agreement with the recommendations of the Gabonese National Ethics Committee (Authorization NPROT/0020/2013I/S G/CNE).

The capture of the rats was carried out from April to September 2018 inside the Makokou Regional Hospital and from the outpatient houses near the hospital. The rodents (rats) were captured using live traps (Tomahawk and Sherman) as described by Duplantier [31]. To capture the rats, traps have been installed from 17 h to 18 h in the small forest of the hospital and in some external houses next to the hospital. All the traps were recovered from 6 : 00 to 7 : 00 a.m. and transported to our laboratory. A cotton swab was turned inside the rat rectum and immediately discharged into 2 ml of a sterile mixture of phosphate buffered saline (PBS) and glycerol (80%/20%) in an Eppendorf tube and was kept at 4°C pending for further analysis.

In the bacteriology laboratory of the International Center for Medical Research of Franceville (CIRMF), each fecal sample was enriched with heart-brain broth (BHB) and streaked on Methylene Blue Eosin ((EMB) (bioMérieux, France) supplemented with 2 mg/L cefotaxime and incubated at 37°C for 24 h. After incubation, each colony, differentiated by structure and color, was picked and transferred by the same means and incubated in the same conditions. The purified colonies were subjected to biochemical identification by using the VITEK® 2 Compact 15 (bioMérieux, Marcy l'étoile, France).

Antibiotic resistance was assessed by the diffusion disc method [32] and inhibition diameters were interpreted using Clinical Laboratory Standard Institute (CLSI) guidelines [33]. Extended-spectrum beta-lactamase production was tested with the double-disc synergy test. The comparative study of the results of a set of beta-lactam antibiotics tested simultaneously on the same antibiogram was carried out to determine the acquired or intrinsic phenotype [34–37].

### 2.2. Determination of Gene Resistance by the Polymerase Chain Reaction

The primer pair SHV-F (5′-3′) and SHV-R (5′-3′) was used from reference [38] and the primer pair CTX- M-F (5′-3′) and CTX-M-R (5′-3′) were used from reference [39] for the characterization of gene resistance.

The amplification of the genes was carried out using a thermal cycler (T100 Thermal Cycler, BIO-RAD). The PCR steps were composed of denaturation for 5 minutes at 94°C, 30 cycles of 30 seconds at 94°C, 30 seconds at 55°C, and 30 seconds at 72°C, and a final extension of 7 min at 72°C. Then, the amplicons were analyzed by agarose gel migration. The revelation was made by previously preparing a 1.5% agarose migration gel stained with ethidium bromide (1 *μ*l/ml) for 30 min under 100 V in 1X TAE buffer and subjected to a 264 nm UV lamp. The software Statistical Package for Social Science (IBM SPSS Statistics 20) was used for the statistical analysis.

## 3. Results

### 3.1. Prevalence of ESBL *E.coli* in Rats

Of 161 feces of rats, all collected in and around the hospital, 32 MBE agar green color colonies were identified as *E. coli* after confirmation and were ESBL-producing *E. coli* with a prevalence of 32/161 (20%).

### 3.2. Prevalence of Resistance to Different Antibiotic Families: Case of Rats

**Figure 1:**
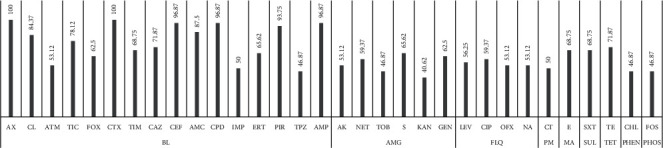
Prevalence of antibiotic resistance. *Y*-axis (%); *X*-axis (antibiotics and families).

As shown in Figure 1, among the antibiotics of the beta-lactam family, the most resistant was amoxicillin, followed cefotaxim and cefepim. In the aminoglycoside family, streptomycin and gentamycin were the most prevalent. In the fluoroquinolone family, ciprofloxacin was the most prevalent. The families of phenicol (chloramphenicol) and phosphonic acid (fosfomycin) were the least resistant.

Aztreonam (TM), Imipenem (IMP), Ertapenem (ERT), Piperacillin-Tazobactam (TPZ), Amakacin (AK), Netilmicin (NET), Tobramycin (TOB), Gentamicin (GEN), Colistin (CT), Tetracycline (TE)), Chloramphenicol (CHL), Fosfomycin (FOS), Levofloxacin (LEV), Ciprofloxacin (CIP), Ofloxacin (OFX), Nalidixic acid (NA), Streptomycin (S), Kanamycin (KAN), Trimethoprim + Sulphamethoxazole (SXT)), Cefoxitin (FOX), Ticarcillin + Clavulanic acid (TIM), Ceftazidime (CAZ), Cefepime (CEF), Erythromycin (E), finally antibiotics considered to be more resistant Amoxicillin (AML), Cephalexin (CL), Ticarcillin (TIC), Cefotaxime (CTX) Amoxicillin + Clavulanic acid (AMC) Cefpodoxime (CPD), Piperacillin (PIR) Ampicillin (AM), Beta-lactam (BL), Aminoglycoside (AMG), Fluoroquinolone-Quinolone (FLQ), Polymixin (PM), Macrolid (MA), Sulfamid (SUL), Tetracyclin (TET), Phenycol (PHEN), and Phosphonic acid (PHOS).

### 3.3. Resistance Genes Identified in Rats

Of the 32 strains of *E. coli* with phenotypic ESBL production, 11 (34.37%) expressed the CTX-M gene.

## 4. Discussion

The purpose of this study was to determine the prevalence of *E*. *coli* producing ESBL in rat feces collected in the town of Makokou.

Mammals such as rats occupying various ecological niches and adapting to different feeding patterns may harbor and contribute to the spread of antimicrobial-resistant bacterial species [39–42]. Several authors worldwide have conducted antimicrobial resistance studies of *E.coli* producing BLSE [43–45].

This study revealed the presence of 20% of *E. coli* producing ESBL in fecal samples of rats. The prevalence of ESBL-producing *E.coli* in rats is similar in the study from Kenya (20%) [27] and from Conakry (Guinea, West Africa) (20%) [13].

The most resistant antibiotic family in rats is beta-lactamines followed by aminoglycosides (netilmycin, amikacin, gentamycin, kanamycin, streptomycin, and tobramycin), tetracyclines, and fluoroquinolones (nalidixic acid, ofloxacin, ciprofloxacin, and levofloxacin). The rats were collected from hospitals and surrounding homes. Third-generation cephalosporins are widely used in hospitals in Gabon and are considered to be responsible for the emergence of extended-spectrum beta-lactamase (ESBL) [42]. These antibiotics (beta-lactam, aminoglycosides, and quinolones) are used in the first line in the treatment of human infections of the bacterial origin [46, 47]. Hence, the prevalence of resistance to urban wild mammals depends on the antibiotics consumed by human populations [48–50]. Imipenem is the molecule that has shown the greatest susceptibility in the majority of bacteria isolated. Its relatively high cost is an advantage, and it reduces the risk of excessive use and, thus, the development of resistance [51]. This may explain the low prevalence of resistance to carbapenems, which are antibiotics used as a last resort in the treatment of infections in human medicine [52].

The prevalences of resistance to streptomycin (65.6%), cefotxime (100%), and tetracycline (71.8%) were higher in our study compared to other studies [15, 27]. The prevalences of resistance to nalidixic acid (53%) and amoxicillin + clavulanic acid (87.5%) in our study were lower compared to those in other studies conducted.

Rats in sewer tunnels showed more resistance than captured rats in other areas of the city, probably because these rats' sewer tunnels were in contact with human sewage [39].

These results showed the presence of CTX-M in rats. ESBL gene type CTX-M-15 is the most common among the isolates of *Enterobacteriaceae* of humans and animals [53, 54]. The spread worldwide of ESBL *Enterobacteriaceae* clinical isolates is a serious problem for the treatment of infectious diseases, in particular the emergence of *E. coli* producing CTX-M-15 [16]. CTX-M-15 is probably the most widespread ESBL gene in humans worldwide [55] and the most detected in human clinical contexts [56]. In addition, the CTX-M-15 and SHV-11 genes are recognized as plasmid-mediated resistance genes [42, 57]. In Gabon, similar studies conducted at the Omar Bongo Ondimba Military Hospital in Libreville and the Albert Schweitzer Hospital in Lambaréné revealed the presence of CTX-M and VHS in hospitalized patients [42, 45]. CTX-M-15 seems to have a particular capacity for dissemination [58]. This could explain the presence of these genes in the population of rats [42, 45].

## 5. Conclusions

This study provided an inventory of antibiotic resistance in the urban wildlife. The presence of multidrug-resistant *E.coli*, in particular those producing ESBL, has been demonstrated in rats that are gregarious mammals, close to humans. As is the case around the world, CTX-M family enzymes predominate, regardless of the bacterial species involved or the compartment in which the gene has been identified. However, these CTX-M genes could be CTX-M-15. In summary, our results show the presence of ESBL-producing *E.coli* from rat population of Makokou. Given the health conditions in this region, rats carrying ESBL-producing Enterobacteria pose a risk to human health and domestic animals.

## Data Availability

Our information is currently restricted. They can be shared at the request of researchers who are interested in our work.

## References

[1] Cattoir V., Bicêtre F. (2008). Les nouvelles bêta-lactamases à spectre étendu (BLSE). *Pathologie Infectieuse en Réanimation*.

[2] Cantón R., Novais A., Valverde A. (2008). Prevalence and spread of extended-spectrum *β*-lactamase-producing *Enterobacteriaceae* in Europe. *Clinical Microbiology and Infection*.

[3] Pehrsson E. C., Forsberg K. J., Gibson M. K., Ahmadi S., Dantas G. (2013). Novel resistance functions uncovered using functional metagenomic investigations of resistance reservoirs. *Frontiers in Microbiology*.

[4] Bradford P. A. (2001). Extended-spectrum *β*-lactamases in the 21st century: characterization, epidemiology, and detection of this important resistance threat. *Clinical Microbiology Reviews*.

[5] Sassa A. M. (2014). Inventaire et prévalence des tiques du bétail dans les élevages de l’Adamaoua au Cameroun. *Revue Africaine de Santé et de Productions Animales*.

[6] Wellington E. M., Boxall A. B., Cross P. (2013). The role of the natural environment in the emergence of antibiotic resistance in Gram-negative bacteria. *The Lancet Infectious Diseases*.

[7] M. J.-M. (2018). OMS: SOS antibiorésistance mondiale. *Revue Francophone des Laboratoires*.

[8] Livermore D. M. (2003). Bacterial resistance: origins, epidemiology, and impact. *Clinical Infectious Diseases*.

[9] Guyomard Rabenirina S. (2016). *Résistance Aux antibiotiques des entérobactéries en guadeloupe: importance en mileu communautaire et diffusion environnementale*.

[10] Goldberg T. L., Gillespie T. R., Rwego I. B., Wheeler E., Estoff E. L., Chapman C. A. (2007). Patterns of gastrointestinal bacterial exchange between chimpanzees and humans involved in research and tourism in western Uganda. *Biological Conservation*.

[11] Mbehang Nguema P. P., Okubo T., Tsuchida S. (2015b). Isolation of multiple drug-resistant enteric bacteria from feces of wild Western Lowland Gorilla (*Gorilla gorilla* gorilla) in Gabon. *Journal of Veterinary Medical Science*.

[12] Miller E. A., Johnson T. J., Omondi G. (2019). Assessing transmission of antimicrobial-resistant *Escherichia coli* in wild giraffe contact networks. *Applied and Environmental Microbiology*.

[13] Schaufler K., Nowak K., Düx A. (2018). Clinically relevant ESBL-producing *K. pneumoniae* ST307 and *E. coli* ST38 in an urban west African rat population. *Frontiers in Microbiology*.

[14] Blyton M. D. J., Pi H., Vangchhia B. (2015). Genetic structure and antimicrobial resistance of *Escherichia coli* and cryptic clades in birds with diverse human associations. *Applied and Environmental Microbiology*.

[15] Graves S., Kennelly-Merrit S. A., Tidemann C. R., Rawlinson P. A., Harvey K. J., Thornton I. W. B. (1988). Antibiotic-resistance patterns of enteric bacteria of wild mammals on the Krakatau Islands and West Java, Indonesia. *Philosophical Transactions of the Royal Society of London. B, Biological Sciences*.

[16] Literak I., Dolejska M., Radimersky T. (2010). Antimicrobial-resistant faecal *Escherichia coli* in wild mammals in central Europe: multi resistant *Escherichia coli* producing extended-spectrum beta-lactamases in wild boars. *Journal of Applied Microbiology*.

[17] Oluduro A. O. (2012). Antibiotic-resistant commensal *Escherichia coli* in faecal droplets from bats and poultry in Nigeria. *Veterinaria Italiana*.

[18] Himsworth C. G., Parsons K. L., Jardine C., Patrick D. M. (2013). Rats, cities, people, and pathogens: a systematic review and narrative synthesis of literature regarding the ecology of rat-associated zoonoses in urban centers. *Vector-Borne and Zoonotic Diseases*.

[19] Meerburg B. G., Singleton G. R., Kijlstra A. (2009). Rodent-borne diseases and their risks for public health. *Critical Reviews in Microbiology*.

[20] Burriel A. R., Kritas S. K., Kontos V. (2008). Some microbiological aspects of rats captured alive at the port city of Piraeus, Greece. *International Journal of Environmental Health Research*.

[21] Desvars-Larrive A., Ruppitsch W., Lepuschitz S. (2019). Urban brown rats (*Rattus norvegicus*) as possible source of multidrug-resistant *Enterobacteriaceae* and meticillin-resistant *Staphylococcus* spp, Vienna, Austria, 2016 and 2017. *Eurosurveillance*.

[22] Gakuya F. M., Kyule M. N., Gathura P. B., Kariuki S. (2001). Antimicrobial resistance of bacterial organisms isolated from rats. *East African Medical Journal*.

[23] Guenther S., Bethe A., Fruth A. (2012). Frequent combination of antimicrobial multiresistance and extraintestinal pathogenicity in *Escherichia coli* isolates from urban rats (*Rattus norvegicus*) in Berlin, Germany. *PLoS One*.

[24] Hansen T. A., Joshi T., Larsen A. R. (2016). Vancomycin gene selection in the microbiome of urbanRattus norvegicusfrom hospital environment. *Evolution, Medicine, and Public Health*.

[25] Kato Y., Matsunaga S.-I., Misuna Y., Ushioda H., Yamamoto T., Kaneuchi C. (1995). Isolation and characterization of *Staphylococcus aureus* in rats trapped at restaurants in buildings in downtown Tokyo. *The Journal of Veterinary Medical Science*.

[26] Gakuya F. M., Kyule M. N., Gathura P. B., Kariuki S. (2001). Antimicrobial susceptibility and plasmids from *Escherichia coli* isolated from rats. *East African Medical Journal*.

[27] Boundenga L., Ngoubangoye B., Ntie S. (2019). Rodent malaria in Gabon: diversity and host range. *International Journal for Parasitology: Parasites and Wildlife*.

[28] Ngoubangoye B., Maganga G. D., Boundenga L. (2019). Absence of paramyxovirus RNA in non-human primate sanctuaries and a primatology center in Gabon. *Journal of Epidemiological Research*.

[29] Ngoubangoye B. L. M. (2017). *Recherche D’agents Infectieux Circulant dans une Communauté d’hôtes, Intérêt Pour la Conservation des PNHs et Risque d’émergence de Maladies Zoonotiquesau Centre De Primatologie du CIRMF et dans les Sanctuaires de PNHs (au Gabon) Rimatologie du CIRMF (Gabon)*.

[30] Duplantier J.-M. (1989). Les rongeurs myomorphes forestiers du nord-est du Gabon: structure du peuplement, démographie, domaines vitaux. *Annual Review of Ecology, Evolution, and Systematics*.

[31] Bauer A. W., Kirby W. M. M., Sherris J. C., Turck M. (1966). Antibiotic susceptibility testing by a standardized single disk method. *American Journal of Clinical Pathology*.

[32] CLSI, Performance Standards for Antimicrobial Susceptibility Testing (2013). Twenty-third informational supplement. *CLSI Document M100-S22*.

[33] Gardien E., Olive C., Chout R., Garcera Y., Jouannelle J. (1997). Les entérobactéries hospitalières en Martinique en 1995: distribution des phénotypes de résistance aux *β*-lactamines de 4 511 souches, urinaires et non urinaires. *Médecine et Maladies Infectieuses*.

[34] Touati A. (2003). Etude des phenotypes de resistance aux *β*-lactamines des souches d’enterobacteries isolees en milieu hospitalier: cas de l’hopital d’amizour (W. Bejaia). *Sciences & Technologie. A, Sciences Exactes*.

[35] Vedel G., Ratovohery D., Paul G., Nevot P. (1994). Phénotypes de Résistance des Entérobactéries aux *β*-lactamines: Description Etdétection.

[36] Vedel G., Peyret M., Gayral J. P., Millot P. (1996). Evaluation of an expert system linked to a rapid antibiotic susceptibility testing system for the detection of *β*-lactam resistance phenotypes. *Research in Microbiology*.

[37] Stürenburg E. (2004). A novel extended-spectrum-lactamase CTX-M-23 with a P167T substitution in the active-site omega loop associated with ceftazidime resistance. *Journal of Antimicrobial Chemotherapy*.

[38] Guenther S., Ewers C., Wieler L. H. (2011). Extended-spectrum beta-lactamases producing *E. coli* in wildlife, yet another form of environmental pollution?. *Frontiers in Microbiology*.

[39] Afiukwa F. N. (2016). First report of blaCTX-M-15 extended spectrum beta-lactamase (ESBL) producing *E. Coli* isolated from cloacal swabs of birds in south Eastern Nigeria. *International Archives of BioMedical and Clinical Research*.

[40] Edelstein M., Pimkin M., Palagin I., Edelstein I., Stratchounski L. (2003). Prevalence and molecular epidemiology of CTX-M extended-spectrum *β*-lactamase-producing *Escherichia coli* and *Klebsiella pneumoniae* in Russian hospitals. *Antimicrobial Agents and Chemotherapy*.

[41] Yala J.-F. (2016). Phenotypic and genotypic characterization of extended-spectrum-beta-lactamases producing-*Enterobacteriaceae* (ESBLE) in patients attending Omar Bongo Ondimba military hospital at Libreville (Gabon). *Current Research in Microbiology and Biotechnology*.

[42] Benavides J. A., Shiva C., Virhuez M. (2018). Extended-spectrum beta-lactamase-producing *Escherichia coli* in common vampire bats *Desmodus rotundus* and livestock in Peru. *Zoonoses and Public Health*.

[43] Bonnet R. (2004). Growing group of extended-spectrum *β*-lactamases: the CTX-M enzymes. *Antimicrobial Agents and Chemotherapy*.

[44] Schaumburg F., Alabi A., Kokou C. (2013). High burden of extended-spectrum *β*-lactamase-producing *Enterobacteriaceae* in Gabon. *Journal of Antimicrobial Chemotherapy*.

[45] Benavides J. A., Godreuil S., Bodenham R. (2012). No Evidence for transmission of antibiotic-resistant *Escherichia coli* strains from humans to wild western lowland gorillas in lopé national park, Gabon. *Applied and Environmental Microbiology*.

[46] Vlieghe E., Phoba M. F., Tamfun J. J. M., Jacobs J. (2009). Antibiotic resistance among bacterial pathogens in Central Africa: a review of the published literature between 1955 and 2008. *International Journal of Antimicrobial Agents*.

[47] Allen S. E., Boerlin P., Janecko N. (2011). Antimicrobial resistance in GenericEscherichia coliIsolates from wild small mammals living in swine farm, residential, landfill, and natural environments in southern ontario, Canada. *Applied and Environmental Microbiology*.

[48] Kozak G. K., Boerlin P., Janecko N., Reid-Smith R. J., Jardine C. (2009). Antimicrobial resistance in *Escherichia coli* isolates from swine and wild small mammals in the proximity of swine farms and in natural environments in ontario, Canada. *Applied and Environmental Microbiology*.

[49] Skurnik D. (2006). Effect of human vicinity on antimicrobial resistance and integrons in animal faecal *Escherichia coli*. *Journal of Antimicrobial Chemotherapy*.

[50] Ndang Ngou Milama S. F. P., Mougougou A., Massandé J. (2019). Étude du profil de sensibilité des bactéries responsables d’infections urinaires communautaires de l’adulte en milieu urologique. *Bulletin Médical d’Owendo*.

[51] Hordijk J., Schoormans A., Kwakernaak M. (2013). High prevalence of fecal carriage of extended spectrum *β*-lactamase/AmpC-producing *Enterobacteriaceae* in cats and dogs. *Frontiers in Microbiology*.

[52] Gonçalves A., Igrejas G., Radhouani H. (2013). Antimicrobial resistance in faecal enterococci andEscherichia coliisolates recovered from Iberian wolf. *Letters in Applied Microbiology*.

[53] Liakopoulos A., Mevius D. J., Olsen B., Bonnedahl J. (2016). The colistin resistancemcr-1gene is going wild: table 1. *Journal of Antimicrobial Chemotherapy*.

[54] Radhouani H., Pinto L., Coelho C. (2009). Detection of *Escherichia coli* harbouring extended-spectrum-lactamases of the CTX-M classes in faecal samples of common buzzards (*Buteo buteo*). *Journal of Antimicrobial Chemotherapy*.

[55] Garcês A., Correia S., Amorim F., Pereira J. E., Igrejas G., Poeta P. (2017). First report on extended-spectrum beta-lactamase (ESBL) producing *Escherichia coli* from European free-tailed bats (Tadarida teniotis) in Portugal: a one-health approach of a hidden contamination problem. *Journal of Hazardous Materials*.

[56] Santos T., Silva N., Igrejas G. (2013). Dissemination of antibiotic resistant *Enterococcus* spp. and *Escherichia coli* from wild birds of Azores Archipelago. *Anaerobe*.

[57] Nicolas-Chanoine M.-H., Blanco J., Leflon-Guibout V. (2008). Intercontinental emergence of *Escherichia coli* clone O25: H4-ST131 producing CTX-M-15. *Journal of Antimicrobial Chemotherapy*.

[58] Nicolas-Chanoine M.-H., Blanco J., Leflon-Guibout V. Intercontinental emergence of Escherichia coli clone O25: H4-ST131 producing CTX-M-15. *Journal of Antimicrobial Chemotherapy*.

